# Blue‐green neutrophilic inclusion bodies in the critically ill patient

**DOI:** 10.1002/ccr3.2196

**Published:** 2019-05-16

**Authors:** Michael P. Soos, Caroline Heideman, Cameron Shumway, Min Cho, Andrew Woolf, Chintalapudi Kumar

**Affiliations:** ^1^ McLaren Greater Lansing Internal Medicine Lansing Michigan; ^2^ McLaren Greater Lansing Gastroenterology Lansing Michigan; ^3^ McLaren Greater Lansing Critical Care Lansing Michigan

**Keywords:** blue‐green neutrophils, cardiac arrest, critically ill, green crystals, shock liver

## Abstract

Previously termed “Green Crystals of Death,” bright blue‐green inclusion bodies within neutrophils are a rare clinical finding. They have been reported as a predictive sign of mortality in critically ill patients and often accompany acute liver failure, lactic acidosis with multisystem organ failure, or septic shock due to *Escherichia coli*.

## 1 INTRODUCTION

Blue‐green neutrophilic inclusion bodies are a rare clinical finding closely associated with morbidity and mortality. Their presence on peripheral smear has been linked with multiple organ failure, especially acute hepatic failure, and septic shock secondary to *Escherichia coli* septicemia. Here, we describe a 61‐year‐old male patient who presented with acute toxic metabolic encephalopathy from an unknown source. Due to hemodynamic instability and increasing blood urea nitrogen (BUN) and creatinine, and indicating signs of renal failure, the patient was transferred to the intensive care unit (ICU) and continuous renal replacement therapy (CRRT) was initiated. The patient's condition worsened and required multiple rounds of advanced cardiac life support (ACLS). A peripheral blood smear revealed green crystals within neutrophils. These inclusion bodies can be released by necrotic hepatocytes in the setting of acute liver failure. Our case supports that blue‐green neutrophilic inclusion bodies in the presence of lactic acidosis and acute liver failure can possibly serve as a predictor of a high mortality rate.

## 2 CASE REPORT

A 61‐year‐old Caucasian male patient presented for concerns of sepsis. He had a medical history of diabetes type II, renal transplant on tacrolimus and mycophenolic acid, chronic kidney disease stage III, heart failure due to ischemic cardiomyopathy, hypertension, atrial fibrillation, and chronic obstructive pulmonary disease. The patient was unable to provide an adequate history at the time of admission due to acute metabolic encephalopathy.

According to documentation provided by emergency medical services, the patient had experienced multiple episodes of emesis and diarrhea. His presenting vitals are available in Table 1. Upon arrival, blood and urine cultures were obtained, a peripheral blood smear ordered and the patient was started on empiric antibiotics with vancomycin and piperacillin/tazobactam. He received 4 L of normal saline. A chest X‐ray (CXR) revealed bilateral pleural effusions consistent with volume overload. A computed tomography (CT) scan of the abdomen and pelvis without contrast revealed moderate right and small left pleural effusions, pancreatic atrophy, renal atrophy, and a right iliac transplanted kidney. An electrocardiogram (ECG) revealed junctional tachycardia with questionable atrial fibrillation. Nephrology was consulted secondary to elevated blood urea nitrogen (BUN) and creatinine and recommended continuous renal replacement therapy (CRRT).

**Table 1:** Presenting vital signs

**Blood pressure**	**Pulse**
107/65 mm Hg	164 bpm
**Respiratory rate**	**Temperature**
40 rpm	102.0°F

The patient was admitted to the Intensive care unit (ICU) where his mentation continued to worsen and he became hypotensive (BP: 91/41). Physical exam revealed bibasilar rhonchi, tachycardia without a murmur, and pitting edema bilaterally. A Foley catheter was placed, and the patient received 40 mg of IV furosemide. He was started on bilevel positive airway pressure (BiPAP) for a deteriorating respiratory status. Overnight, the patient became increasingly bradycardic and developed asystole. ACLS protocols were performed resulting in the return of spontaneous circulation (ROSC). The patient required three additional rounds of ACLS for a total cumulative code time of approximately one hour. Following ROSC, the patient was intubated without sedation. The patient was started on norepinephrine and vasopressin continuous infusions for hemodynamic support. Later that morning, a central venous catheter was placed in the left internal jugular vein and the patient was started on epinephrine and bicarbonate continuous infusions.

Postarrest laboratory results, which can be seen in Table 2, revealed lactic acidosis with a pH of 7.135 and peak lactic acid of 9.5 mg/dL. A complete metabolic panel revealed elevated transaminases with peak aspartate aminotransferase (AST) >1600 U/L and alanine aminotransferase (ALT) of 3490 U/L consistent with hepatic shock. Sedation weaning trials the following morning revealed that the patient was tolerating mechanical ventilation without sedation. At that time, neurology was consulted secondary to suspicion for anoxic brain injury. Examination of the patient's lower extremities revealed the presence of bullae and erythema. Due to a lack of clinical improvement in empiric antibiotic therapy, the infectious disease service was consulted.

**Table 2:** Laboratory results by hospital day

Hospital day	One	Four	Five	Six
Bilirubin (mg/dL)	4.4	5.8	7.9	9.0
AST (unit/L)	>1600	913	360	144
ALT (unit/L)	>3500	2899	2039	1229
Alkaline phosphatase (unit/L)	67	131	184	235
BUN (mg/dL)	58	33	34	37
Creatinine (mg/dL)	3.02	1.74	1.52	1.45
WBC (K/µL)	19.1	28.5	16.9	9.1
Hemoglobin (g/dL)	6.2	9.3	8.9	8.3
Platelets (K/µL)	96	69	28	18
Potassium (mmol/L)	6.6	4.8	4.8	5.6
eGFR (mL/min)	26	40	47	49
Lactic Acid (mg/dL^3^)	9.5	2.9	1.6	1.7

Abbreviation(s): ALT, alanine aminotransferase; AST, aspartate aminotransferase; BUN, blood urea nitrogen; eGFR, estimated glomerular filtration rate; WBC, white blood cell.

**Figure 1:**
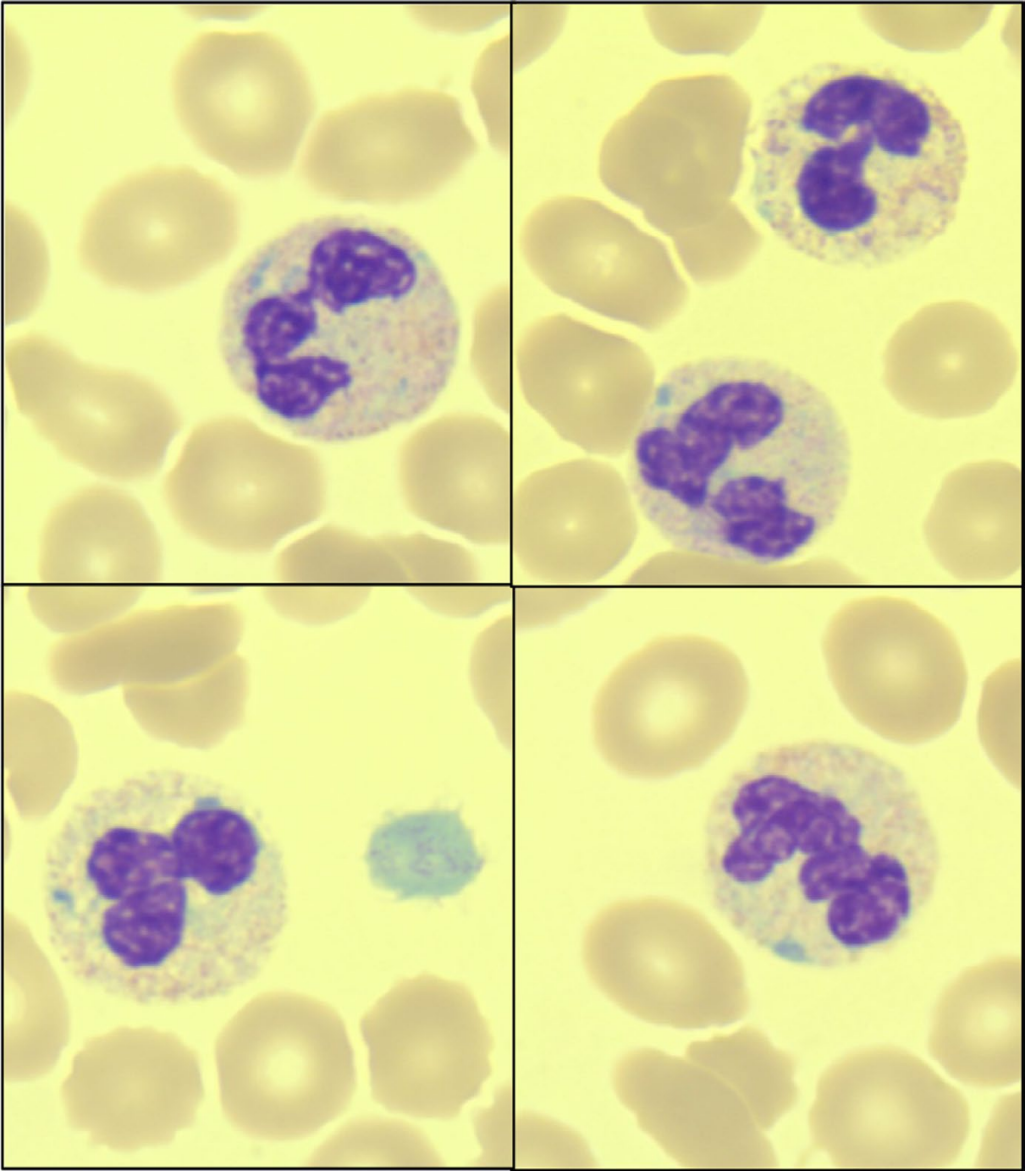
Photographs taken via digital microscopy showing the presence of multiple blue‐green inclusion bodies in various neutrophils throughout the patient's peripheral blood smear

On day three, the findings of the peripheral blood smear were finalized which revealed pancytopenia with macrocytic anemia. It was noted that a rare neutrophil contained a few green crystals as seen in Figure 1. The pathologist noted that these findings could be seen in patients with critical illnesses in the face of acute hepatic failure. These inclusion bodies are also known as “green crystals of hematology.” The patient's case was discussed with the pathology department and further review of the slide revealed multiple neutrophils with similar findings described above.

On the fourth day, the patient's lactic acidosis and transaminases improved (Lactic acid: 1.7 mg/dL^3^, AST: 144 U/L, ALT: 1229 U/L); however, the patient showed no clinical change. Blood and urine cultures showed no growth to date. A neurologist evaluated the patient and ordered an electroencephalogram (EEG) revealing findings consistent with anoxic brain injury. Three physicians reviewed and discussed the patient's case and determined that further care was futile. The correctional facility was contacted, and the patient was made comfort care with the agreement of the warden and next of kin. The patient expired later that morning.

## 3 DISCUSSION

Blue‐green inclusion bodies found within the cytoplasm of neutrophils are a rare clinical finding. When present, these inclusion bodies have been identified as a heralding sign for increased risk of morbidity and mortality. Current literature has acknowledged an association between acute hepatic failure and these green neutrophilic bodies. The risks of morbidity and mortality are further increased when lactic acidosis is present.^1^ In reported cases with the presence of lactic acidosis, death typically occurs between 48 and 72 hours after discovery on a peripheral blood smear. The findings on the smear should be considered a critical finding and should be reported as quickly as possible.

The pathogenesis of the blue‐green inclusion bodies is not well understood at this time. Current hypotheses suggest that the inclusions likely originate from lipofuscin. Lipofuscin is a name given to the yellow‐pigmented granules comprised of lipid‐containing residues of lysosomal digestion. Lipofuscin is considered a “wear‐and‐tear” pigment found in the liver, heart, kidney, retina, nerve cells, and the adrenal glands. In the currently hypothesized pathogenesis, acute hepatic failure secondary to hypoxic injury results in the release of lipofuscin from necrotic hepatic parenchymal cells. Macrophages and Kupffer cells then take up the pigmented granules resulting in the formation of blue‐green inclusion bodies.^2^ Most commonly, these are seen in neutrophils, but may occasionally be found in monocytes. Some other studies claim that the inclusions may be secondary to blood‐borne bile products.^3^

The inclusions are most often associated with acute liver failure, lactic acidosis with multisystem organ failure secondary to trauma, and *E coli*‐associated septic shock. The largest and most current study done by Hodgson et al suggested that their presence is strongly associated with ischemic liver injury, typically occurring in the context of shock or liver transplantation. Elevated liver transaminases and gamma‐glutamyl transferase (GGT) were documented in a majority of cases, with a mean peak AST of 2900 U/L, ALT of 2024 U/L, and GGT of 156 U/L. This case supports the hypothesis that it is associated with liver pathology as the patients AST and ALT peaked at >1600 and 3490 U/L, respectively. In our study, blood cultures drawn at admission showed no growth at 96 hours, which would suggest the patient likely suffered an ischemic hepatic injury due to prolonged ACLS leading to fulminant liver failure.

The presence of lactic acidosis was another finding that correlated with the presence of the blue‐green inclusions, which was also shown in our patient's case. This most likely characterizes a mixture of higher oxygen demand, poor tissue perfusion, and rapid loss of lactate dehydrogenase secondary to hepatic necrosis. In previous studies, 93% of patients showed elevated lactate levels on arterial blood gases (ABG). In this case, the patient's lactic acid peaked at 9.5 mg/dL^3^. In available studies, when lactic acidosis was present with recorded lactate of >5 mg/dL^3^,^4^, ^5^ the mortality rate was 100%. Our case supports the current literature published and serves as a reminder to clinicians that the presence of blue‐green inclusion bodies on the peripheral smear in conjunction with lactic acidosis can be used as a predictor of mortality in critically ill patients with hepatic failure.

## CONFLICT OF INTEREST

None declared.

## AUTHOR CONTRIBUTIONS

MPS and CH: served as the primary authors; CS: served as the secondary author; MC and AW: served as the secondary authors and were responsible for literature review; CK: served as the senior author.
